# Visceral obesity augments prescription use: An analysis of the cross-sectional study of NHANES 2011–2018

**DOI:** 10.1371/journal.pone.0318413

**Published:** 2025-02-03

**Authors:** Maximino Acevedo-Fernández, Renata Ochoa Precoma, Leonardo M. Porchia, Victor M. Posadas, Enrique Torres-Rasgado, M. Elba Gonzalez-Mejia, Esther López-Bayghen

**Affiliations:** 1 Departamento de Genética, Facultad de Medicina, Benemérita Universidad Autónoma de Puebla, Puebla, México; 2 Instituto de Infertilidad y Genética México SC, Instituto Ingenes, México City, México; 3 Departamento de Toxicología, Centro de Investigación y de Estudios Avanzados del Instituto Politécnico Nacional (CINVESTAV-IPN), México City, México; Instituto Nacional de Cardiologia Ignacio Chavez, MEXICO

## Abstract

**Background:**

Visceral obesity (VATob) increases the risk for many diseases. Central obesity has been associated with an augmented prescription use; however, there is a paucity of research focused on VATob. Here, the aim was to evaluate the association between VATob and prescription use.

**Methods:**

Data was collected from the NHANES dataset (2011–2018). Visceral adipose tissue was measured using dual x-ray absorptiometry, and VATob was defined as ≥100 cm^2^. Prescription use was collected from the RXQ_RX files and classified according to Vademecum. Association between VATob and prescription use was determined with logistic regression and reported as odds ratios (ORs) with 95% confidence intervals (95%CIs).

**Results:**

10,952 participants (weighted: 121,090,702) were included, in which 41.8% were VATob and 52.0% of them had ≥1 prescription. Overall, VATob demonstrated an augmented rate of prescription use when compared to non-VATob (52.0% versus 36.7%, p<0.001), specifically with metabolic (4.5-fold increase), cardiovascular (3.9-fold increase), gastrointestinal (2.5-fold increase), and hematopoietic agents (2.3-fold increase). This was associated with increased the risk for overall prescription use (OR_overall_ = 1.9, 95%CI: 1.7–2.1, p<0.001). Similar results were observed with metabolic and cardiovascular agents. However, when stratified by BMI, normal weight participants (OR_metabolic_ = 10.4; 95%CI: 6.5–16.6 & OR_cardiovascular_ = 7.0; 95%CI: 4.4–11.1, p<0.001) had a greater risk than the overweight (OR_metabolic_ = 4.1; 95%CI: 3.0–5.6 & OR_cardiovascular_ = 3.4; 95%CI: 2.5–4.7, p<0.001) or obese participants (OR_metabolic_ = 3.5; 95%CI: 2.3–5.3 & OR_cardiovascular_ = 3.5; 95%CI: 2.5–4.9, p<0.001).

**Conclusion:**

VATob is associated with augmented prescription use, particularly with cardiovascular and metabolic agents. This association was higher for normal weight participants.

## Introduction

Prescription use has increased dramatically over the past 50 years. In the United States of America (USA), from 2009 to 2022, the annual number of prescriptions rose from 4.0 billion to 6.7 billion [1], equating to about 19 prescriptions per citizen. Per capita spending increased from around $200 USD in the early 1980s to between $1000 and $1200 USD by 2015 and has remained constant. Since 2009, the cost of brand-name prescription drugs has doubled [2]. Improper dosage (overdosing and underdosing), inappropriate prescribing (targeting and selection), and inadequate monitoring have contributed to an increase in adverse drug effects and drug interactions [3]. Additionally, some clinicians prescribe more medications to counter these adverse effects. Hoel *et al*. suggest that polypharmacy (5 or more medications) is associated with increased hospital admissions, adverse drug events, non-adherence, and higher healthcare costs [4]. Consequently, prescription use has become a significant health concern, necessitating efforts to address its root causes.

Several factors contribute to the rise in prescription use, including an aging population, the prioritization of medications as the first-line of treatment (specially for chronic non-communicable diseases), medicalization, prolong medication use without discontinuation, and the increasing prevalence of modifiable co-factors, such as obesity. Among these, obesity is one of the most modifiable contributors to increased prescription use.

Over the years, augmented prescription use has been linked to obesity. Kantor *et al*. associated this trend with the rise in obesity prevalence [5]. Obesity—a complex, multifactorial disease—is characterized by the growth of bioactive adipose tissues that promote chronic diseases and reduce life expectancy [6]. Since 1980, global obesity prevalence has doubled, with almost 30% of the population now classified as overweight or obese [7]. If current trends persist, it is projected that 60% of the population will be overweight or obese by 2030 [8], likely driving further increases in prescription use.

In clinical practice, obesity is commonly assessed using the body mass index (BMI), which reflects general obesity, comprising both visceral adipose tissue (VAT) and subcutaneous adipose tissue (SAT). Importantly, different types of SAT have varying effects on disease risk. For instance, abdominal SAT is linked to metabolic diseases, whereas femoral-gluteal SAT has a neutral effect [9]. Thus, BMI may be a weak predictor of certain pathologies. Studies suggest that abdominal adipose tissue correlates more strongly with metabolic-associated steatotic liver disease (MASLD), insulin resistance, and other metabolic diseases than BMI [10–12]. Consequently, abdominal adipose tissue might serve as a better indicator for evaluating association with prescription use [7, 13].

VAT is particularly metabolically active compared to SAT, producing adipokines that augment metabolic and cardiovascular risk [14]. VAT is the adipose tissue that is posited around the organs in the peritoneal cavity and visceral obesity (VATob), also known as “Hidden Obesity”, is a threshold that is associated with an increased risk for certain diseases [15]. Numerous studies have demonstrated that VATob increases the risk of dyslipidemia, Type 2 Diabetes, hypertension, cardiovascular disease, and other metabolic abnormalities [16–18], potentially increasing prescription use. Interestingly, VATob in individuals with normal BMI (18.5–24.9 kg/m^2^) has been linked to a higher risk of MASLD than in obese individuals with VATob (BMI range: 30.0–39.9 kg/m^2^) [19]. However, measuring VAT is not routine in clinical practice, leaving many VATob cases undiagnosed across BMI categories.

Since most obesity-related complications require pharmaceutical intervention as part of the treatment [20], it is posited that the association between VAT and prescription use parallels that observed with general obesity [21].

Here, the aim was to evaluate the association between VATob and prescription use using data from the National Health and Nutrition Examination Survey (NHANES).

## Materials and methods

### Data source

This study was conducted following the Strengthening the Reporting of Observational Studies in Epidemiology (STROBE) guidelines (S1 Table). Data were obtained from the publicly available NHANES dataset, administered and managed by the National Center for Health Statistics (NCHS) [22]. The data was downloaded and compiled on March 2, 2023. Due to the NCHS policies, all participant identities remained anonymous to the authors. To have a nationally representative survey of the health and nutrition status of the USA, the NCHS uses a stratified multistage probability model. The NHANES received its ethical approval for all study protocols from the NCHS Research Ethics Review Board [23]. All participants provided written consent by signing a comprehensive informed consent [23]. All procedures followed by the NHANES were in accordance with the Declaration of Helsinki of 1975.

This study utilized data from 2011 to 2018. To be part of this study, the participants had to 1) have values of VAT, as measured by whole-body dual-energy x-ray absorptiometry (available for individuals aged 8 to 59 years) and 2) be ≥18 years old. The participants were excluded if they had the following conditions: 1) missing BMI values or were <18.5 kg/m^2^, or ≥40 kg/m^2^; 2) were pregnant; 3) suffered from viral diseases or potential infections (HIV, Hepatitis B, B/D, or C); or 4) had cancer. These exclusion criteria were chosen because the listed conditions could independently increase prescription use through mechanisms unrelated to adipose tissue.

### Measurement methods and instrumentation

Data was collected according to the standardized protocols for the NHANES [24]. Three classes of variables were collected: anthropometric measurements, demographic data, and laboratory tests. The anthropometric variables obtained were height (cm), weight (kg), BMI (kg/m^2^), waist circumference (cm), systolic and diastolic blood pressure (SBP and DBP, mmHg), and VAT (cm^2^). VAT was categorized using a cutoff value of 100 cm^2^ into VATob or non-VATob [25]. According to the World Health Organization, BMI was classified into normal weight (18.5–24.9 kg/m^2^), overweight (25.0–29.9 kg/m^2^), obese class 1 (30.0–34.9 kg/m^2^), or obese class 2 (35.0–39.9 kg/m^2^). Hypertension was defined as SBP ≥ 130 mmHg or DBP ≥90 mmHg.

Demographic data included biological sex (male and female), age (years), and ethnicity (Non-Hispanic Black, Non-Hispanic White, Mexican American, other Hispanics, or other races), education level [<9th grade, 9-11th grade (including 12th grade with no diploma), High School graduate/GED or equivalent, some college or associate degree, college graduate or above], marital status (Single, Married, Divorced/Separated, Widowed), income (<$19.9k, $20-$74.9k, $75-$99.9k, ≥$100k) and physical activity. For physical activity, the metabolic equivalent (MET) was calculated using data from the Global Physical Activity Questionnaire [26]. This questionnaire uses 2 components: types of daily activities (work, recreational, walking/biking) and intensity (moderate and vigorous). The data collected was the frequency of the activity and the duration spent per month or week. To calculate the MET-minutes/week, vigorous-intensity activities were assigned as 8.0 MET, whereas moderate-intensity activities were assigned as 4.0 MET. For each participant, each MET category was calculated by multiplying the number of days by the average time by the corresponding MET modifier. Total MET score was estimated by summing up 1) vigorous work activity/vigorous recreational activities, 2) moderate work activity/moderate recreational activities, and 3) walking/bicycling. MET scores was classified as low (<600 MET-minutes/week), moderate (600–2999 MET-minutes/week), and high (≥3000 MET-minutes/week). Insurance data was also obtained: 1) if the participants were covered by insurance; 2) if they had private insurance, and 3) if their plans covered medications.

During the interview, the survey participants were asked if they are currently using prescription medications. Those who answered affirmatively provided information concerning all prescription medications, in which the containers were examined and medication names were recorded, duration, and reasons for use. Prescription data were obtained from RXQ_RX files and classified using Vademecum [27] into 1) cardiovascular, 2) metabolic agents, 3) gastrointestinal, 4) central nervous system, 5) psychotherapeutic, 6) respiratory, 7) hematopoietic, 8) topical nasal, 9) antineoplastic, 10) anti-infective, 11) hormones/hormone modifiers, 12) topical, 13) ophthalmic, or 14) otic agents.

The laboratory variables collected were total cholesterol (mg/dL), high-density lipoprotein cholesterol (mg/dL), and glycosylated hemoglobin (HbA1c, %). According to the American Diabetes Association criteria, subjects were classified as either normal glucose tolerance (HbA1c: <5.6%), prediabetics (HbA1c: 5.6–6.4%) and Type 2 Diabetics (HbA1c: ≥6.5%). The laboratory variables were only reported if the missing data for a variable was <5%.

### Statistical analysis

All statistical analyses were performed using the Statistical Package for the Social Sciences software version 26.0 (SPSS, IBM Corp., Armonk, NY, USA). The complex samples design option was used to account for NHANES’s stratified multistage probability sampling design. For quantitative and categorical variables, the mean or the percentage, respectively, with standard errors were calculated. For differences between the groups, the Rao Scott-Chi^2^ test was used for categorical variables, whereas the designed-adjusted T-test was used for continuous variables. The association between VAT and prescription use was determined with univariate and multivariate logistic regression and reported as odds ratios (ORs) with 95% confidence intervals (95%CIs). P-values were considered significant if they were <0.05 (two-tailed). The participants were classified in to 4 groups based on 2 characteristics: 1) VATob and 2) Prescription use. VATob was defined as VAT ≥100 cm^2^. Positive prescription use was defined as participants taking one or more medications. The 4 groups are 1) the control group: without VATob and without prescription use (non-VATob/RX-), 2) the prescription only group (non-VATob/RX+), 3) the VATob only group (VATob/RX-), and 4) the VATob who are taking a prescription group (VATob/RX+). For the complete cohort, participants were included if data was present for VAT and prescription use. Other variables were shown only if <5% of the data was missing. When selecting variables to be adjusted, if the sample size decreased by >10% as well as the portions between the independent and dependent variables changed by >1%, then the variable would not be included.

## Results

### Selection of participants

**Fig 1:**
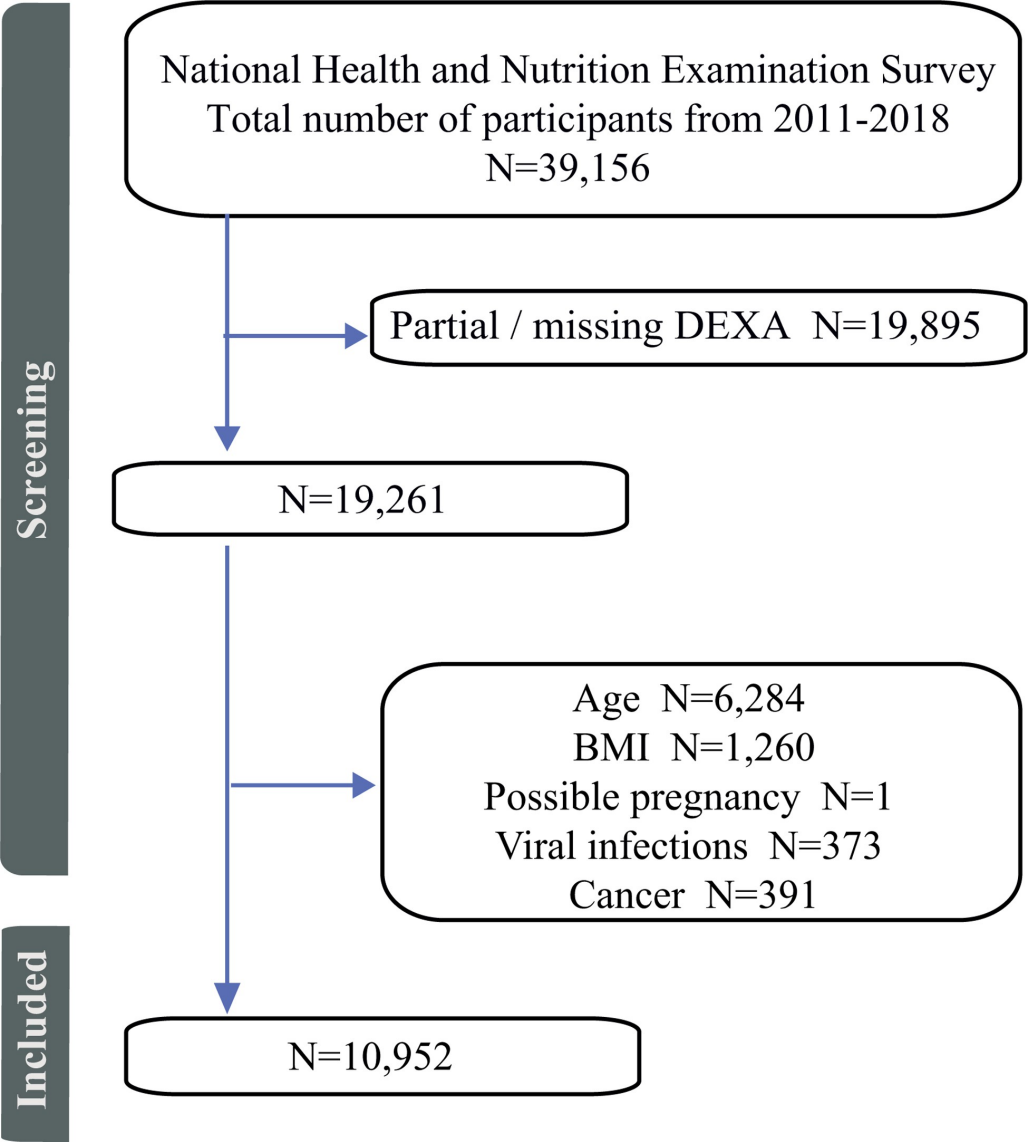
Flow chart of the screening process for selecting eligible participants to assess the effect visceral obesity has on prescription use.

Of the 39,156 potential participants, 49.2% had acceptable DEXA scans; however, 8,308 records were removed due to age, being underweight or morbidly obese, or having a condition that could affect adiposity (Fig 1). This resulted in 10,952 participants (weighted: 121,090,702) included in this study. The characteristics of the cohort are shown in Table 1.

**Table 1:** Characteristics of participants included in the study, stratified by VATob.

Category	Non-VATob		VATob	
Number of prescriptions	0	≥1	0	≥1
N ‐ crude	4546	2063	2268	2075
N ‐ representative	44,613,987	25,820,728	24,315,833	26,340,154
*Demographic variables*				
Sex ‐ Female (%)	43.5 ± 0.7 ^b, c^	66.6 ± 1.3 ^a, c, d^	34.2 ± 1.2 ^a, b, d^	44.8 ± 1.5 ^b, c^
Age (years)	32.2 ± 0.3 ^b, c, d^	36.7 ± 0.4 ^a, c, d^	41.2 ± 0.4 ^a, b, d^	47.1 ± 0.3 ^a, b, c^
Ethnicity (%)				
White	52.7 ± 2.0 ^b, d^	70.4 ± 2.0 ^a, c^	55.7 ± 2.6 ^b, d^	70.1 ± 1.9 ^a, c^
Black	15.7 ± 1.3 ^c, d^	12.4 ± 1.2 ^c, d^	6.6 ± 0.7 ^a, b^	7.7 ± 0.8 ^a, b^
Mexican-American	10.5 ± 1.2 ^b, c^	4.5 ± 0.6 ^a, c, d^	19.9 ± 1.9 ^a, b, d^	7.8 ± 1.0 ^b, c^
Other Hispanic	8.9 ± 0.9 ^b, d^	4.7 ± 0.6 ^a, c^	9.6 ± 1.0 ^b, d^	5.7 ± 0.7 ^a, c^
Other races	12.1 ± 0.8 ^b, c, d^	8.0 ± 0.7 ^a^	8.2 ± 0.8 ^a^	8.7 ± 0.9 ^a^
Marital status (%)				
Married/Cohabitating	52.8 ± 1.4 ^c, d^	54.4 ± 1.8 ^c, d^	68.8 ± 1.5 ^a, b^	69.0 ± 1.2 ^a, b^
Single	31.6 ± 1.3 ^c, d^	27.6 ± 1.6 ^c, d^	15.9 ± 1.1 ^a, b, d^	12.9 ± 1.0 ^a, b, c^
Divorced/Separated	6.5 ± 0.4 ^b, c, d^	11.3 ± 0.9 ^a, d^	12.8 ± 1.1 ^a, d^	16.0 ± 1.1 ^a, b, c^
Widowed	0.4 ± 0.1 ^c, d^	1.0 ± 0.3	1.3 ± 0.4 ^a^	1.8 ± 0.3 ^a^
Not reported	8.7 ± 0.5 ^b, c, d^	5.7 ± 0.6 ^a, c, d^	1.2 ± 0.2 ^a, b, d^	0.3 ± 0.1 ^a, b, c^
Education level (%)				
College graduate	31.8 ± 1.6 ^b, c^	39.5 ± 1.9 ^a, c, d^	23.4 ± 1.7 ^a, b, d^	30.0 ± 2.2 ^b, c^
Some college	29.0 ± 1.0 ^d^	30.4 ± 1.8 ^d^	31.3 ± 1.4	35.1 ± 1.5 ^a, b^
High school graduated	19.1 ± 0.9 ^b, c, d^	16.3 ± 1.3 ^c, d^	24.7 ± 1.3 ^a, b^	23.0 ± 1.2 ^a, b^
Some high school	8.2 ± 0.6 ^c^	6.7 ± 0.7 ^c^	12.3 ± 1.0 ^a, b, d^	7.9 ± 0.8 ^c^
9^th^ grade or below	3.2 ± 0.4 ^b, c^	1.4 ± 0.3 ^a, c, d^	7.1 ± 0.8 ^a, b, d^	3.7 ± 0.5 ^b, c^
Not reported	8.7 ± 0.5 ^b, c, d^	5.7 ± 0.6 ^a, c, d^	1.2 ± 0.2 ^a, b, d^	0.3 ± 0.1 ^a, b, c^
Income (%)				
≥100K	25.3 ± 1.5 ^b, d^	34.3 ± 2.0 ^a, c^	22.0 ± 2.1 ^b, d^	31.8 ± 2.2 ^a, c^
75K-99.9K	11.4 ± 0.8	12.5 ± 0.9	12.4 ± 1.2	13.3 ± 1.2
20K-74.9K	44.2 ± 1.5 ^b, c^	38.5 ± 1.8 ^a, c^	49.8 ± 2.0 ^a, b, d^	42.2 ± 1.5 ^c^
0–19.9K	13.4 ± 1.0 ^d^	11.6 ± 1.2	11.9 ± 0.9	9.6 ± 0.9 ^a^
Not reported	5.7 ± 0.5 ^b, c, d^	3.1 ± 0.5 ^a^	4.0 ± 0.6 ^a^	3.2 ± 0.5 ^a^
Insurance (%)				
Covered by insurance	74.3 ± 1.2 ^b, c, d^	89.3 ± 1.1 ^a, c^	67.7 ± 1.6 ^a, b, d^	90.3 ± 0.8 ^a, c^
Private insurance	77.9 ± 1.2	78.1 ± 1.3	80.5 ± 1.6	77.6 ± 1.5
Plan covers prescription	92.9 ± 0.6 ^b, d^	96.8 ± 0.5 ^a, c^	92.9 ± 0.9 ^b, d^	96.0 ± 0.7 ^a, c^
Physical Activity ‐ MET-minutes/week	5547 ± 204 ^b, d^	4331 ± 154 ^a, d^	4766 ± 216 ^a, d^	3233 ± 167 ^a, b, c^
Low: <600	20.9 ± 0.8 ^c, d^	23.5 ± 1.3 ^c, d^	30.2 ± 1.5 ^a, b^	37.9 ± 1.3 ^a, b^
Moderate: 600–2999	30.4 ± 1.1 ^b, d^	36.9 ± 1.6 ^a, c^	29.5 ± 1.6 ^b, d^	35.0 ± 1.4 ^a, c^
High: ≥3000	45.1 ± 1.1 ^b, c, d^	39.7 ± 1.5 ^a, d^	40.4 ± 1.7 ^a, d^	27.0 ± 1.4 ^a, b, c^
*Anthropometric variables*				
Weight (kg)	73.5 ± 0.3 ^b, c, d^	71.7 ± 0.4 ^a, c, d^	90.4 ± 0.5 ^a, b^	91.2 ± 0.5 ^a, b^
Height (cm)	169.9 ± 0.2 ^b^	168.0 ± 0.3 ^a, c, d^	170.5 ± 0.3 ^b^	170.4 ± 0.4 ^b^
Waist circumference (cm)	87.8 ± 0.2 ^c, d^	88.4 ± 0.4 ^c, d^	105.2 ± 0.3 ^a, b, d^	106.9 ± 0.4 ^a, b, c^
Body Mass Index (kg/m^2^)	25.4 ± 0.1 ^c, d^	25.3 ± 0.1 ^c, d^	31.0 ± 0.1 ^a, b^	31.4 ± 0.2 ^a, b^
Normal-weight (%)	51.5 ± 1.1 ^c, d^	53.4 ± 1.6 ^c, d^	5.6 ± 0.8 ^a, b^	5.8 ± 0.7 ^a, b^
Overweight (%)	34.1 ± 1.0 ^c^	33.0 ± 1.5 ^c^	38.9 ± 1.2 ^a, b, d^	32.7 ± 1.5 ^c^
Obesity class I (%)	11.8 ± 0.8 ^c, d^	10.3 ± 0.9 ^c, d^	36.7 ± 1.3 ^a, b^	40.4 ± 1.5 ^a, b^
Obesity class II (%)	2.7 ± 0.3 ^c, d^	3.3 ± 0.5 ^c, d^	18.9 ± 1.0 ^a, b^	21.1 ± 1.3 ^a, b^
Systolic blood pressure (mmHg)	116.7 ± 0.3 ^c, d^	117.5 ± 0.5 ^c, d^	124.0 ± 0.4 ^a, b^	125.0 ± 0.4 ^a, b^
Diastolic blood pressure (mmHg)	68.1 ± 0.3 ^b, c, d^	70.2 ± 0.3 ^a, c, d^	75.4 ± 0.4 ^a, b^	75.9 ± 0.3 ^a, b^
American Heart Association Classification				
Normal (%)	88.6 ± 0.7 ^b, c, d^	84.3 ± 1.0 ^a, c, d^	71.2 ± 1.4 ^a, b^	68.4 ± 1.4 ^a, b^
Elevated (%)	9.2 ± 0.6 ^b, c, d^	12.5 ± 1.0 ^a, c, d^	21.2 ± 1.1 ^a, b^	24.1 ± 1.3 ^a, b^
Stage I (%)	1.6 ± 0.2 ^b, c, d^	2.7 ± 0.5 ^a, c, d^	5.3 ± 0.7 ^a, b^	6.6 ± 0.8 ^a, b^
Stage II (%)	0.6 ± 0.1 ^c^	0.5 ± 0.2 ^c^	2.3 ± 0.5 ^a, b, d^	0.9 ± 0.2 ^c^
Total abdominal adipose tissue area (cm^2^)	307.1 ± 3.4 ^b, c, d^	340.5 ± 4.3 ^a, c, d^	523.5 ± 4.2 ^a, b, d^	553.3 ± 5.1 ^a, b, c^
VAT (cm^2^)	60.6 ± 0.6 ^c, d^	61.2 ± 0.8 ^c, d^	141.5 ± 1.0 ^a, b, d^	154.3 ± 1.3 ^a, b, c^
SAT (cm^2^)	246.6 ± 3.0 ^b, c, d^	279.3 ± 3.7 ^a, c, d^	382.0 ± 3.7 ^a, b, d^	399.0 ± 4.3 ^a, b, c^
*Laboratory variables*				
HbA1c (%)	5.3 ± 0.1 ^b, c, d^	5.4 ± 0.1 ^a, c, d^	5.6 ± 0.1 ^a, b, d^	5.9 ± 0.1 ^a, b, c^
American Diabetes Association Classification				
Normal glucose tolerance	93.6 ± 0.5 ^b, c, d^	90.3 ± 1.1 ^a, c, d^	80.0 ± 1.2 ^a, b, d^	66.4 ± 1.3 ^a, b, c^
Prediabetes	5.8 ± 0.5 ^c, d^	6.7 ± 0.8 ^c, d^	16.2 ± 1.0 ^a, b, d^	20.2 ± 1.1 ^a, b, c^
Diabetes	0.5 ± 0.1 ^b, c, d^	3.0 ± 0.4 ^a, d^	3.9 ± 0.5 ^a, d^	13.3 ± 1.2 ^a, b, c^
Total cholesterol (mg/dL)	179.2 ± 0.7 ^b, c, d^	186.0 ± 1.2 ^a, c, d^	204.4 ± 1.3 ^a, b^	201.3 ± 1.3 ^a, b^
High-density lipoprotein (mg/dL)	55.8 ± 0.4 ^b, c, d^	59.5 ± 0.5 ^a, c, d^	46.4 ± 0.4 ^a, b, d^	48.3 ± 0.4 ^a, b, c^

Data are either mean or frequency ± standard error. Group differences between categorical data were calculated Rao-Scott Chi-square Test, whereas group differences between continuous data were calculated by using the General Linear Model. P-values <0.05 (two-tailed) were considered significant.

^a^ vs non-VATob / no prescriptions.

^b^ vs non-VATob / has at least 1 prescription.

^c^ vs VATob / no prescriptions.

^d^ vs VATob / has at least 1 prescription.

Overall, the VATob rate was 41.8±1.0%, with 47.4±1.3% of males and 35.5±1.2% of females having VATob. Interestingly, 7.3±0.8% of normal-weight participants had VATob, compared to 43.2±1.4%, 71.2±1.4%, and 83.4±1.5% of overweight, obese class 1, and obese class 2 participants, respectively. When stratified by VATob and prescription use, 36.8% of the cohort was classified as non-VATob/RX-, whereas 20.1% of the cohort was VATob/RX-, 21.3% were non-VATob/RX+, and 21.8% were VATob/RX+. Significant differences were observed among the four group with respect to race, marital status, education level, income, and physical activity. The principal ethnicity for all groups was Non-Hispanic Whites. Non-Hispanic Blacks were the second most prevalent for the non-VATob groups, while Mexican Americans were more prevalent in the VATob groups. As expected, the VATob groups were older with elevated weight, waist circumference, BMI (specifically the prevalence of overweight and obese classes 1 and 2), SAT, systolic and diastolic blood pressure, HbA1c, and total cholesterol levels. Additionally, the prevalence of hypertension, prediabetes, and Type 2 Diabetes was higher among the VATob groups. For each group, >67% had some form of health insurance, with private insurance being the most common form. Of participants with insurance, >90% of the insurance plans had prescription coverage.

### VATob effect on prescription use

**Fig 2:**
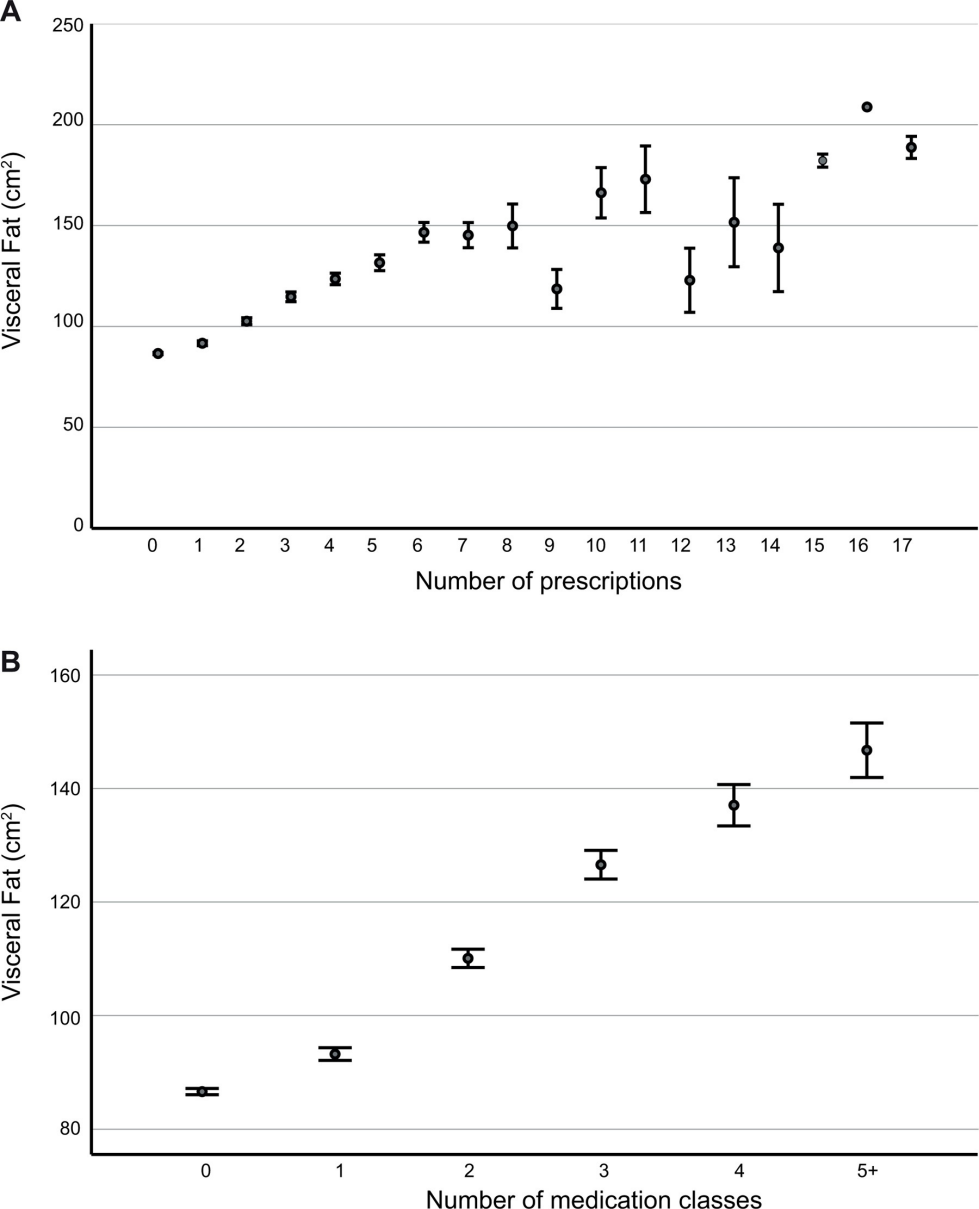
Visceral adipose tissue (VAT) affects prescription use. Means (dot) and standard error (bars) plots were constructed the determine the relationship between VAT and number of prescriptions (A) and the number of medication classes (B).

The number of prescriptions among participants ranged from none to 17, with approximately 0.6% reporting the use of 10 or more prescriptions. A significant positive correlation was observed between VAT and the number of prescriptions (r = 0.281, p<0.001, Fig 2A). Concerning medicine classes, 1 person was taking 10 different kinds of medication, none were taking 9 different classes, 7 were taking 8 different classes, 20 were taking 7 different classes, 52 were taking 6 different classes, and 114 were taking 5 different classes. Due to the low reporting rate, ≥5 was grouped together. VAT also significantly correlated with the number of medication classes (r = 0.263, p<0.001, Fig 2B).

When VATob was considered, participants with VATob had a higher rate of prescription use compared to those without VATob (52.0±1.0% vs. 36.7±0.9%, p<0.001, Table 2). When stratified by BMI class, the proportion of VATob users taking at least one medication was significantly higher in the VATob group than in the non-VATob group for normal-weight (53.1±4.9% versus 37.5±1.2%, respectively, p = 0.002), for overweight (47.6±1.7% versus 35.9±1.8%, respectively, p<0.001), for obese class 1 (54.4±1.6% versus 33.7±2.7%, respectively, p<0.001), for obese class 2 (54.8±2.1% versus 41.6±3.5%, respectively, p = 0.002). When stratified by the number of prescriptions, the non-VATob group was less likely to be taking ≥2 medications than the VATob group (17.1±0.7% versus 34.5±1.0%, respectively, p<0.001), and similarly, they were less likely to be taking ≥5 medications (3.0±0.3% versus 9.7±0.6%, respectively, p<0.001).

**Table 2:** Frequencies of drug usage between VATob and non-VATob groups.

Prescription drugs	Non-VATob (%)	VATob (%)	p-value
Overall	36.7 ± 0.9	52.0 ± 1.0	<0.001*
Cardiovascular agents	6.0 ± 0.4	23.4 ± 1.0	<0.001*
Metabolic agents	4.2 ± 0.4	19.0 ± 0.8	<0.001*
Gastrointestinal agents	4.0 ± 0.4	10.1 ± 0.6	<0.001*
Central nervous system agents	9.8 ± 0.6	14.6 ± 0.8	<0.001*
Psychotherapeutic agents	9.7 ± 0.6	13.7 ± 0.8	<0.001*
Respiratory agents	4.6 ± 0.3	6.0 ± 0.5	0.013*
Hematopoietic agents	0.7 ± 0.1	1.6 ± 0.2	0.001*
Topical nasal agents	1.4 ± 0.2	2.3 ± 0.3	0.022*
Antineoplastic agents	0.7 ± 0.1	0.7 ± 0.2	0.761
Anti-infective agents	4.8 ± 0.4	4.1 ± 0.4	0.217
Hormones/hormone modifiers	12.8 ± 0.7	12.9 ± 0.7	0.880
Topical agents	1.5 ± 0.2	1.3 ± 0.2	0.616
Ophthalmic agents	0.6 ± 0.1	0.7 ± 0.2	0.417
Otic agents	0.1 ± 0.1	0.1 ± 0.1	0.791

Values are presented with frequency (%) ± standard error.

**Fig 3:**
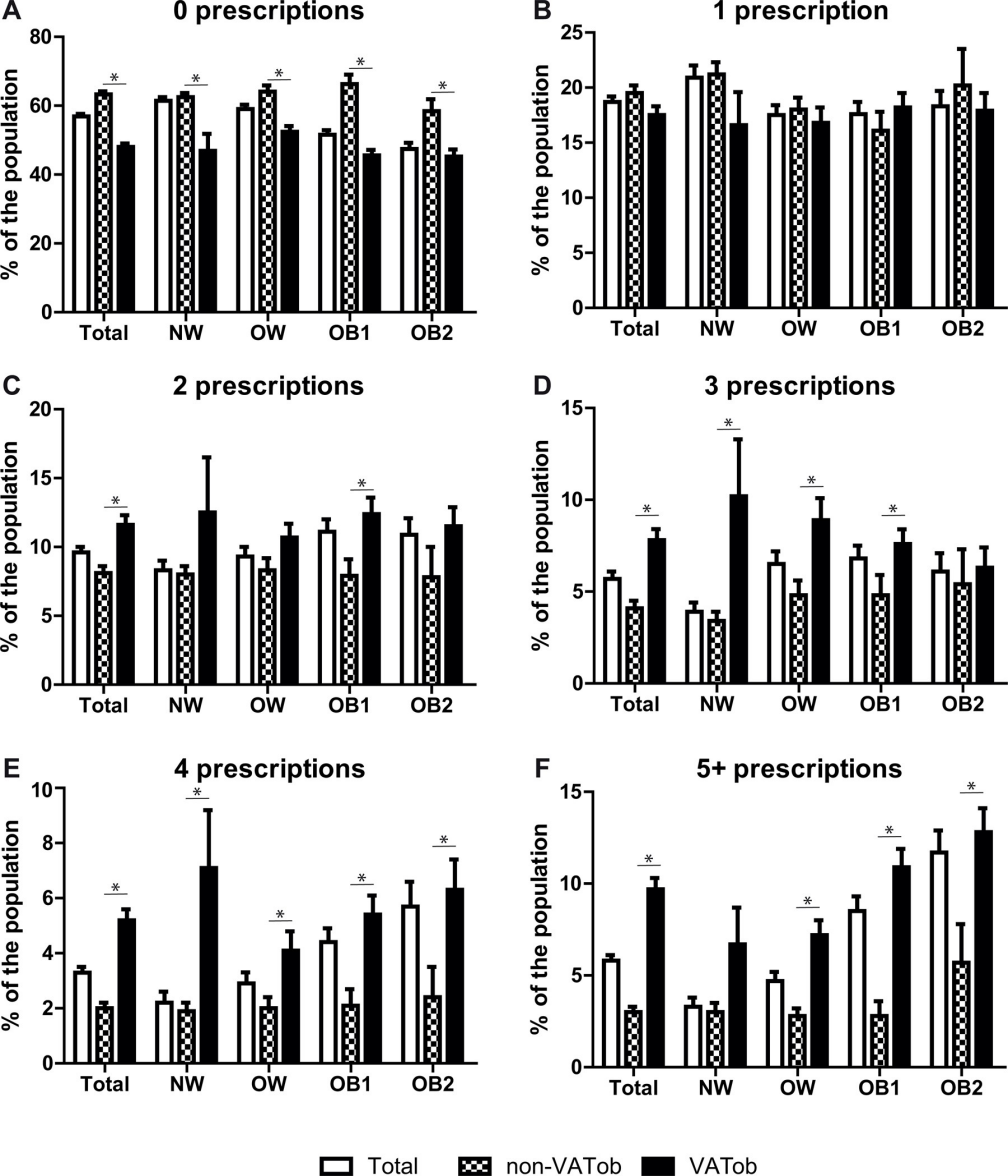
The effect of visceral obesity (VATob) has on prescription use. Bar charts were constructed for the percentage who were taking 0 (A), 1 (B), 2 (C), 3 (D), 4 (E), or ≥5 (F) prescriptions. For each panel, the rate of use for the complete cohort (Total, white bar) or when stratified into non-VATob (checkered bar) or VATob (black bar) was determined for the total cohort (Total) or when stratified by BMI class: normal weight (NW), overweight (OW), obese class 1 (OB1), and obese class 2 (OB2). * Indicates a significant difference (p<0.05) between non-VATob and VATob group.

Further stratification by general obesity revealed a significant difference in the distribution of the number of prescriptions used by VATob participants according to BMI class (p<0.001). For normal weight participants, VATob was associated with increased use of 3 and 4 prescriptions (p<0.05, Fig 3) and showed a near-significant association with the use of ≥5 medications (p = 0.073). For overweight participants, VATob was associated with increased use of 3, 4, and ≥5 prescriptions (p<0.05). For obese class 1 participants, VATob was associated with increased use of 2, 3, 4, and ≥5 prescriptions (p<0.05). For obese class 2 participants, VATob was associated with increased use of 4 and ≥5 prescriptions (p<0.05). No effect was observed for the use of only 1 prescription.

**Fig 4:**
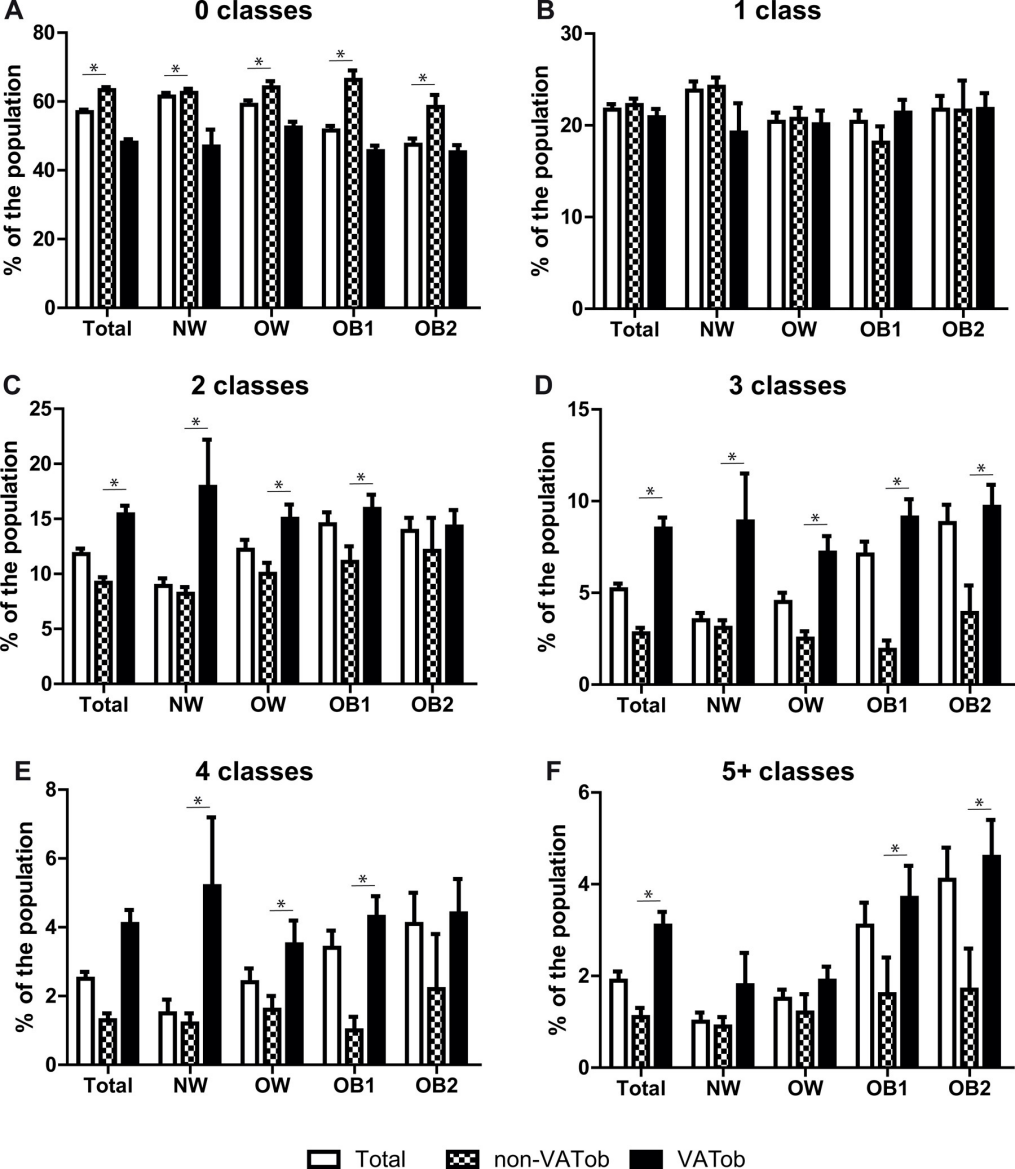
The effect of visceral obesity (VATob) has on the number of medication classes taken. Bar charts were constructed for the percentage who were taking 0 (A), 1 (B), 2 (C), 3 (D), 4 (E), or ≥5 (F) different medication classes. For each panel, the rate of use for the complete cohort (Total, white bar) or when stratified into non-VATob (checkered bar) or VATob (black bar) was determined for the total cohort (Total) or when stratified by BMI class: normal weight (NW), overweight (OW), obese class 1 (OB1), and obese class 2 (OB2). * Indicates a significant difference (p<0.05) between non-VATob and VATob group.

For the number of medication classes, participants in the non-VATob group were less likely to take ≥2 different classes of medications compared to those in the VATob group (14.4±0.6% versus 31.1±1.0%, respectively, p<0.001). Similarly, the non-VATob group was less likely to take ≥5 different classes of medications (1.1±0.2% versus 3.1±0.3%, respectively, p<0.001). When stratified by general obesity, there was significant difference in the distribution medicine classes between VATob and non-VATob participants based on BMI class (p<0.001). For normal-weight and overweight participants, VATob was associated with an increased prevalence of taking medications from 2, 3, and 4 different classes (p<0.05, Fig 4). For obese class 1 participants, VATob was associated with an increased prevalence of taking medications from 2, 3, 4 and ≥5 different classes (p<0.05). For obese class 2 participants, VATob was associated with an increased prevalence of taking medications from 3 and ≥5 different classes (p<0.05). No effect was observed for 1 medication class.

With respect to the type of medication, the most commonly prescribe overall were cardiovascular agents (13.3±0.5%), metabolic agents (10.4±0.4%), central nervous system agents (11.8±0.5%), gastrointestinal agents (6.5±0.4%), anti-infective agents (12.8±0.6%), and psychotherapeutics agents (11.4±0.5%). When VATob status was considered, significant associations were observed with several medication classes. VATob was strongly associated with the use of metabolic agents (4.5-fold), cardiovascular agents (3.9-fold), gastrointestinal agents (2.5-fold), hematopoietic agents (2.3-fold), topical nasal agents (1.6-fold), central nervous system agents (1.5-fold), psychotherapeutic agents (1.4-fold), and respiratory agents (1.3-fold, Table 2).

### VATob increases the risk of prescription use

Potential confounding variables associated with prescription use were evaluated (Table 3). Univariate logistic regression was employed to identify variables that showed an association with prescription use, and multivariate logistic regression was used to confirm these associations while controlling for potential confounders. Age, sex, race, education, marital status, insurance coverage, and BMI were all significantly associated with augmented prescription use. To account for these confounders, all analyses will control for sex and age (Model 1) as well as race, education, marital status, and insurance coverage (Model 2). The impact of general obesity (BMI) will be further examined through stratification.

**Table 3:** Confounding variables for prescribed drug use.

Variable	Univariate	Multivariate (Model 2)
Age (per 10 years)	1.59 (1.51–1.69), <0.001*	1.65 (1.53–1.77), <0.001*
Sex		
Male	1.00 (Referent)	1.00 (Referent)
Female	1.86 (1.71–2.02), <0.001*	1.87 (1.71–2.04), <0.001*
Race		
Non-Hispanic White	1.00 (Referent)	1.00 (Referent)
Mexican American	0.34 (0.29–0.40), <0.001*	0.52 (0.45–0.60), <0.001*
Other Hispanic	0.44 (0.37–0.51), <0.001*	0.54 (0.46–0.63), <0.001*
Non-Hispanic Black	0.62 (0.54–0.70), <0.001*	0.65 (0.57–0.74), <0.001*
Other Races	0.60 (0.50–0.71), <0.001*	0.63 (0.53–0.75), <0.001*
Education		
College graduate	1.00 (Referent)	1.00 (Referent)
Some college	0.91 (0.79–1.06), 0.217	1.14 (0.98–1.34), 0.905
High school	0.78 (0.66–0.91), 0.002*	1.09 (0.92–1.28), 0.416
9-11^th^	0.63 (0.52–0.78), <0.001*	1.08 (0.89–1.31), 0.332
<9^th^	0.47 (0.35–0.62), <0.001*	0.98 (0.72–1.34), 0.098
Not reported	0.41 (0.33–0.51), <0.001*	1.64 (1.23–2.17), 0.001*
Marital status		
Married/Cohabitating	1.00 (Referent)	1.00 (Referent)
Single	0.73 (0.64–0.84), <0.001*	1.43 (1.23–1.65), <0.001*
Divorced/Separated	1.47 (1.24–1.75), <0.001*	1.30 (1.10–1.54), 0.002*
Widowed	1.85 (1.01–3.40), 0.048*	1.15 (0.59–2.23), 0.681
Not reported	0.46 (0.37–0.58), <0.001*	1.00 (1.00–1.00), 0.999
Income (USD$)		
≥100k	1.00 (Referent)	1.00 (Referent)
75k–99.9k	0.80 (0.62–1.03), 0.085	0.98 (0.78–1.26), 0.933
20k–74.9k	0.64 (0.54–0.75), <0.001*	0.90 (0.74–1.09), 0.256
≤19.9k	0.60 (0.49–0.74), <0.001*	0.99 (0.78–1.26), 0.866
Not reported	0.45 (0.35–0.58), <0.001*	0.73 (0.58–0.92), 0.008*
Insurance ‐ Covered	3.44 (2.98–3.98), <0.001*	2.93 (2.42–3.30), <0.001*
BMI category		
Normal weight	1.00 (Referent)	-^a^
Overweight	1.10 (0.97–1.25), 0.120	- ^a^
Obese class I	1.49 (1.29–1.73), <0.001*	- ^a^
Obese class II	1.76 (1.49–2.09), <0.001*	-^a^

Values are odds ratio, 95% confidence interval, p-value. Values were calculated using complex samples design adjusted logistic regression. * indicates a significant result (p<0.05, two-tailed).

^a^ Variable was not included for Model 2.

The effect VATob has on prescription use is shown in Table 4. In the crude analysis, VATob was associated with increase overall prescription use, particularly for cardiovascular, metabolic, gastrointestinal, central nervous system, psychotherapeutic, respiratory, hematopoietic, and topical nasal agents. After controlling for sex, age, race, marital status, education, income, and insurance coverage (Model 2), significant associations persisted for overall prescription use, and the use of cardiovascular, metabolic, gastrointestinal, and psychotherapeutic agents. The interaction between VATob and general obesity status was also evaluated. Significant interactions were observed for cardiovascular (p_interaction_ = 0.026), metabolic (p_interaction_ = 0.003), psychotherapeutic (p_interaction_ = 0.050), and hematopoietic agents (p_interaction_<0.001). When the cohort was stratified by BMI class, a decreasing trend in odds ratios was noted for cardiovascular and metabolic agents, with normal weight participants showing the highest odds ratio. However, after adjustment using Model 2, significant interactions between VATob and general obesity status remained only for metabolic (p_interaction_ = 0.020) and hematopoietic agents (p_interaction_<0.001).

**Table 4:** Visceral obesity effect on prescription usage stratified by body-mass index category.

Group	Non-VATob^a^	VATob^a^	Crude	Model 1^b^	Model 2^c^
*Overall Treatment*					
Complete cohort	4546 / 2063	2268 / 2075	1.87 (1.68–2.08), <0.001*	1.39 (1.23–1.57), <0.001*	1.54 (1.35–1.76), <0.001*
Normal weight	2385 / 1042	143 / 141	1.88 (1.26–2.82), 0.003*	1.57 (1.03–2.39), 0.035*	1.77 (1.11–2.80), 0.017*
Overweight	1477 / 669	878 / 659	1.62 (1.34–1.97), <0.001*	1.10 (0.88–1.37), 0.401	1.22 (0.95–1.56), 0.122
Obese class I	532 / 264	819 / 793	2.35 (1.75–3.15), <0.001*	1.39 (1.02–1.91), 0.039*	1.70 (1.17–2.48), 0.006*
Obese class II	152 / 88	428 / 482	1.71 (1.22–2.38), 0.002*	1.09 (0.76–1.58), 0.631	1.09 (0.70–1.71), 0.689
p_interaction_^d^			0.177	0.129	0.152
*Cardiovascular agents*					
Complete cohort	6187 / 422	3332 / 1011	4.78 (3.95–5.77), <0.001*	2.55 (2.08–3.12), <0.001*	2.79 (2.24–3.48), <0.001*
Normal weight	3267 / 160	218 / 66	6.97 (4.40–11.06), <0.001*	2.80 (1.75–4.49), <0.001*	3.15 (1.88–5.29), <0.001*
Overweight	1992 / 154	1233 / 304	3.40 (2.45–4.72), <0.001*	1.40 (0.99–1.98), 0.059	1.66 (1.13–2.42), 0.010*
Obese class I	718 / 78	1217 / 395	3.81 (2.58–5.62), <0.001*	1.91 (1.25–2.91), 0.003*	2.57 (1.64–4.01), <0.001*
Obese class II	210 / 30	664 / 246	2.54 (1.54–4.20), <0.001*	1.07 (0.58–1.98), 0.829	1.34 (0.69–2.58), 0.381
p_interaction_^d^			0.026*	0.120	0.254
*Metabolic agents*					
Complete cohort	6331 / 278	3517 / 826	5.37 (4.30–6.70), <0.001*	2.43 (1.94–3.03), <0.001*	2.52 (2.02–3.13), <0.001*
Normal weight	3326 / 101	213 / 71	10.41 (6.53–16.60), <0.001*	2.97 (1.83–4.81), <0.001*	3.24 (1.99–5.26), <0.001*
Overweight	2035 / 111	284 / 1268	4.06 (2.96–5.55), <0.001*	1.57 (1.13–2.16), 0.007*	1.69 (1.19–2.39), 0.004*
Obese class I	748 / 48	1297 / 315	3.82 (2.29–6.38), <0.001*	1.53 (0.89–2.65), 0.123	1.73 (0.98–3.05), 0.059
Obese class II	222 / 18	171 / 910	2.71 (1.47–5.01), 0.002*	0.97 (0.46–2.04), 0.926	1.05 (0.51–2.15), 0.897
p_interaction_^d^			0.003*	0.027*	0.020*
*Gastrointestinal agents*					
Complete cohort	6383 / 226	3937 / 406	2.71 (2.12–3.48), <0.001*	1.74 (1.34–2.26), <0.001*	1.74 (1.33–2.28), <0.001*
Normal weight	3318 / 109	260 / 24	2.30 (1.23–4.32), 0.010*	1.11 (0.59–2.11), 0.736	1.09 (0.57–2.08), 0.803
Overweight	2069 / 77	1409 / 128	2.21 (1.49–3.26), <0.001*	1.34 (0.85–2.11), 0.199	1.33 (0.83–2.13), 0.231
Obese class I	776 / 30	1447 / 165	2.83 (1.65–4.87), <0.001*	1.82 (1.07–3.08), 0.027*	1.80 (1.01–3.20), 0.046*
Obese class II	230 / 10	89 / 910	3.48 (1.48–8.18), 0.005*	2.13 (0.89–5.08), 0.089	2.57 (1.09–6.03), 0.031*
p_interaction_^d^			0.787	0.642	0.701
*Central nervous system agents*					
Complete cohort	6104 / 505	3768 / 575	1.58 (1.37–1.82), <0.001*	1.23 (1.02–1.47), 0.029*	1.18 (0.97–1.43), 0.099
Normal weight	3165 / 262	252 / 32	1.63 (0.97–2.73), 0.062	0.99 (0.56–1.76), 0.974	1.00 (0.54–1.85), 0.989
Overweight	1987 / 159	1372 / 165	1.44 (1.08–1.92), 0.013*	1.16 (0.80–1.67), 0.430	1.13 (0.76–1.67), 0.543
Obese class I	739 / 57	1382 / 230	2.21 (1.49–3.29), <0.001*	1.58 (1.04–2.41), 0.033*	1.60 (1.04–2.44), 0.032*
Obese class II	213 / 27	1612 / 762	1.09 (0.58–2.04), 0.795	0.85 (0.44–1.65), 0.620	0.73 (0.35–1.52), 0.395
p_interaction_^d^			0.122	0.120	0.306
*Psychotherapeutic agents*					
Complete cohort	6138 / 471	3862 / 481	1.48 (1.27–1.72), <0.001*	1.34 (1.10–1.63), 0.005*	1.36 (1.10–1.68), 0.006*
Normal weight	3184 / 243	267 / 17	0.89 (0.46–1.71), 0.713	0.75 (0.37–1.51), 0.408	0.75 (0.35–1.64), 0.471
Overweight	1993 / 153	1403 / 134	1.18 (0.86–1.61), 0.294	1.05 (0.71–1.55), 0.806	1.00 (0.65–1.55), 0.992
Obese class I	742 / 54	1402 / 210	2.21 (1.49–3.26), <0.001*	2.05 (1.33–3.14), 0.001*	2.37 (1.44–3.92), 0.001*
Obese class II	219 / 21	790 / 120	1.52 (0.81–2.90), 0.188	1.12 (0.53–2.36), 0.765	1.00 (0.41–2.42), 0.993
p_interaction_^d^			0.050*	0.044*	0.132
*Respiratory agents*					
Complete cohort	6343 / 266	4091 / 252	1.32 (1.06–1.63), 0.013*	1.20 (0.91–1.58), 0.196	1.29 (0.96–1.74), 0.092
Normal weight	3309 / 118	266 / 18	1.39 (0.72–2.68), 0.319	1.12 (0.51–2.47), 0.768	1.29 (0.55–3.02), 0.547
Overweight	2061 / 85	1468 / 69	1.00 (0.71–1.40), 0.996	1.00 (0.61–1.63), 0.988	1.15 (0.68–1.95), 0.602
Obese class I	749 / 47	1518 / 94	1.22 (0.73–2.04), 0.440	0.94 (0.55–1.60), 0.820	0.99 (0.54–1.80), 0.967
Obese class II	224 / 16	839 / 71	1.28 (0.57–2.87), 0.540	1.13 (0.51–2.50), 0.764	1.15 (0.50–2.64) 0.735
p_interaction_^d^			0.820	0.807	0.713
*Hematopoietic agents*					
Complete cohort	6564 / 45	4268 / 75	2.31 (1.44–3.71), 0.001*	1.16 (0.68–1.97), 0.591	1.19 (0.69–2.02), 0.530
Normal weight	3407 / 20	276 / 8	5.38 (1.53–19.62), 0.010*	1.52 (0.41–5.67), 0.526	1.95 (0.51–7.47), 0.326
Overweight	2129 / 17	1521 / 16	0.66 (0.29–1.50), 0.313	0.40 (0.17–0.93), 0.034*	0.38 (0.16–0.88), 0.025*
Obese class I	788 / 8	1575 / 37	2.28 (0.81–6.42), 0.116	1.01 (0.8–3.73), 0.985	0.89 (0.19–4.22), 0.881
Obese class II	240 / 0	896 / 14	Not determined^e^	Not determined^e^	Not determined^e^
p_interaction_^d^			<0.001*	<0.001*	<0.001*
*Topical nasal agents*					
Complete cohort	6531 / 78	4272 / 71	1.63 (1.07–2.47), 0.023*	1.30 (0.81–2.10), 0.272	1.59 (0.96–2.64), 0.074
Normal weight	3391 / 36	278 / 6	3.14 (1.06–9.30), 0.039*	2.12 (0.76–5.96), 0.151	3.23 (1.00–10.50), 0.051
Overweight	2118 / 28	1520 / 17	0.85 (0.35–2.07), 0.710	0.71 (0.25–2.00), 0.510	0.77 (0.26–2.24), 0.620
Obese class I	784 / 12	1579 / 33	2.01 (0.90–4.48), 0.086	1.65 (0.72–3.78), 0.230	2.07 (0.81–5.33), 0.129
Obese class II	238 / 2	895 / 15	1.32 (0.31–5.74), 0.704	0.96 (0.24–3.95), 0.957	0.45 (0.08–2.60), 0.365
p_interaction_^d^			0.238	0.237	0.170

Values are odds ratio (95% confidence interval), p-value. Values were calculated using complex samples design adjusted logistic regression.

* indicates a significant result (p<0.05, two-tailed).

^a^ Number of participants with and without prescriptions

^b^ Model 1: Crude + Sex and age.

^c^ Model 2: Model 1 + Race, Marital status, Education level, Income, and covered by insurance.

^d^ An interaction between VATob and BMI classes was determined by adding an interaction term to the complete cohort model.

^e^ Odds ratio was not calculated due to zero participants in one of the cells.

### Physical activity influences the association between VATob and prescription use

Given the role of physical activity in reducing VAT, its potential mitigating effects on VATob’s association with prescription use were assessed (Table 5). A significant interaction was observed for overall prescription use in the Crude analysis as well as in Model 1 and Model 2. Participants who were highly active exhibited a lower risk of overall prescription use compared to those with lower levels of physical activity. For specific medication classes, however, no significant differences were detected among participants with low, moderate, or high physical activity levels.

**Table 5:** Visceral obesity’s effect on prescription usage stratified by physical activity.

Group	Non-VATob ^a^	VATob^a^	Crude	Model 1^b^	Model 2^c^
*Overall Treatment*					
Low	1085 / 575	759 / 837	2.10 (1.75–2.52), <0.001*	1.49 (1.22–1.81), <0.001*	1.50 (1.19–1.90), 0.001*
Moderate	1451 / 723	637 / 656	2.05 (1.69–2.49), <0.001*	1.62 (1.29–2.03), <0.001*	1.83 (1.42–2.35), <0.001*
High	2010 / 765	872 / 582	1.42 (1.21–1.67), <0.001*	1.11 (0.93–1.32), 0.239	1.24 (1.03–1.50), 0.023*
p_interaction_^d^			0.001*	<0.001*	0.001*
*Cardiovascular agents*					
Low	1516 / 144	1163 / 433	4.98 (3.72–6.68), <0.001*	2.75 (1.99–3.80), <0.001*	3.03 (2.17–4.25), <0.001*
Moderate	2028 / 146	989 / 304	4.29 (3.29–5.60), <0.001*	2.49 (1.80–3.43), <0.001*	2.63 (1.89–3.66), <0.001*
High	2643 / 132	1180 / 274	4.50 (3.29–6.15), <0.001*	2.32 (1.63–3.29), <0.001*	2.84 (1.91–4.21), <0.001*
p_interaction_^d^			0.709	0.630	0.593
*Metabolic agents*					
Low	1566 / 94	1245 / 351	5.00 (3.59–6.96), <0.001*	2.32 (1.61–3.35), <0.001*	2.31 (1.59–3.35), <0.001*
Moderate	2073 / 101	1020 / 273	5.48 (3.81–7.87), <0.001*	2.61 (1.77–3.83), <0.001*	2.70 (1.81–4.02), <0.001*
High	2692 / 83	1252 / 202	4.74 (3.39–6.64), <0.001*	2.07 (1.45–2.94), <0.001*	2.12 (1.46–3.09), <0.001*
p_interaction_^d^			0.840	0.521	0.525
*Gastrointestinal agents*					
Low	1580 / 80	1416 / 180	2.54 (1.72–3.75), <0.001*	1.63 (1.06–2.49), 0.026*	1.51 (0.98–2.23), 0.063
Moderate	2108 / 66	1183 / 110	2.68 (1.72–4.19), <0.001*	1.75 (1.14–2.68), 0.012*	1.75 (1.14–2.68), 0.011*
High	2695 / 80	1338 / 116	2.57 (1.82–3.65), <0.001*	1.75 (1.18–2.59), 0.006*	1.80 (1.18–2.75), 0.007*
p_interaction_^d^			0.978	0.865	0.746
*Central nervous system agents*					
Low	1486 / 174	1327 / 269	1.48 (1.13–1.94), 0.006*	1.09 (0.81–1.47), 0.574	0.97 (0.70–1.34), 0.853
Moderate	2032 / 142	1147 / 146	1.61 (1.25–2.08), <0.001*	1.35 (0.97–1.86), 0.074	1.26 (0.92–1.73), 0.152
High	2586 / 189	1294 / 160	1.36 (1.05–1.75), 0.019*	1.10 (0.81–1.48), 0.539	1.05 (0.77–1.45), 0.738
p_interaction_^d^			0.699	0.461	0.477
*Psychotherapeutic agents*					
Low	1505 / 155	1372 / 224	1.48 (1.16–1.90), 0.002*	1.17 (0.85–1.59), 0.329	1.06 (0.75–1.49), 0.750
Moderate	2017 / 157	1156 / 137	1.52 (1.10–2.10), 0.012*	1.46 (0.98–2.18), 0.062	1.53 (1.00–2.34), 0.050
High	2616 / 159	1334 / 120	1.16 (0.88–1.53), 0.287	1.21 (0.89–1.65), 0.219	1.20 (0.86–1.67), 0.291
p_interaction_^d^			0.354	0.223	0.435
*Respiratory agents*					
Low	1578 / 82	1489 / 107	1.07 (0.70–1.63), 0.753	0.95 (0.61–1.48), 0.822	0.97 (0.61–1.54), 0.885
Moderate	2094 / 80	1213 / 80	1.95 (1.19–3.18), 0.009*	1.80 (0.94–3.47), 0.078	1.99 (1.00–3.98), 0.051
High	2671 / 104	1389 / 65	0.94 (0.61–1.44), 0.771	0.89 (0.56–1.41), 0.612	0.92 (0.56–1.53), 0.751
p_interaction_^d^			0.080	0.053	0.061
*Hematopoietic agents*					
Low	1645 / 15	1561 / 35	2.08 (0.93–4.64), 0.074	1.20 (0.54–2.63), 0.651	1.09 (0.49–2.44), 0.829
Moderate	2157 / 17	1270 / 23	1.85 (0.84–4.10), 0.127	1.06 (0.43–2.60), 0.901	1.07 (0.43–2.65), 0.881
High	2762 / 13	1437 / 17	2.84 (0.94–8.57), 0.063	1.22 (0.35–4.26), 0.747	1.39 (0.44–4.40), 0.569
p_interaction_^d^			0.849	0.892	0.862
*Topical nasal agents*					
Low	1634 / 26	1564 / 32	1.81 (0.94–3.51), 0.076	1.44 (0.68–3.05), 0.334	1.60 (0.69–3.69), 0.269
Moderate	2144 / 30	1267 / 26	1.51 (0.72–3.19), 0.274	1.37 (0.62–3.01), 0.427	1.65 (0.71–3.81), 0.240
High	2753 / 22	1441 / 13	1.27 (0.52–3.15), 0.595	0.95 (0.37–2.42), 0.912	1.27 (0.48–3.37), 0.629
p_interaction_^d^			0.813	0.784	0.957

Values are odds ratio (95% confidence interval), p-value. Values were calculated using complex samples design adjusted logistic regression.

* indicates a significant result (p<0.05, two-tailed).

^a^ Number of participants with and without prescriptions

^b^ Model 1: Crude + Sex, age.

^c^ Model 2: Model 1 + Race, Marital status, Education level, Income, covered by insurance.

^d^ An interaction between VATob and activity level was determined by adding an interaction term to the overall model.

## Discussion

Here, VATob was associated with increased prescription use. Among normal-weight participants, VATob increased the likelihood of using 3 or 4 prescriptions. In contrast, among obese participants, VATob increased the likelihood of using of 2 or more prescriptions. There was also a strong association between VATob and the use of cardiovascular and metabolic agents, especially among normal-weight participants.

VATob is an underestimated pathology, with 27–46% of the population affected [28]. Additionally, many individuals are unaware of the VATob status, particularly normal-weight individuals, where the prevalence is 10–25% [28–30]. In our cohort, 41.8% of participants had VATob, including 7.3% of the normal-weight participants. Regarding biological sex, VATob prevalence varies significantly depending on age and BMI, ranging from 22.8% to 96.6% in men and from 3.5% to 61.5% in women, findings that closely support the results of our study [28]. Therefore, our cohort was similar to other published studies with respect to the rate of VATob by biological sex and BMI class.

Prescription use patterns in our cohort showed that 38% of participants were taking at least one medication, lower than the 64% reported by Barrett *et al*. [31]. Their study, which used BMI to define obesity, found that 64% of obese participants were taking at least 1 medication [31]. Here, 48–53% of obese participants were taking at least 1 medication. Even though both studies used the NHANES with overlapping collection periods, the major difference is the age range. The Barrett *et al*. study used BMI to determine overall obesity, which was collected for all ages. Here, visceral obesity was collected by DEXA, which was collected for participants aged between 8 and 59 years. The exclusion of older participants likely skewed prescription use rates downward. Nonetheless, there are no studies in which the VATob and prescription use are evaluated.

Obesity significantly affects prescription use. The Counterweight Project demonstrated that as BMI categories increased, there was a corresponding rise in prescription use. Both the total number of prescriptions and the variety of medication classes were significantly higher among individuals in the obese category [21]. A study conducted in the United Kingdom reported a 16% increase in prescription use among obese individuals [21], whereas a study in the USA found an 11.3% increase [31]. These findings highlight the influence of obesity on prescription use despite the differing healthcare systems in these two countries. Here, VATob was associated with a 15.3% increase in the overall prescription use. However, stratifying by BMI class demonstrated no observable trend or differences between each class (Table 4), suggesting that VATob has no promoting effect when compared to general obesity.

When analyzing specific medication classes, VATob was associated with augment used of cardiovascular (17.4%), metabolic (14.8%), gastrointestinal (6.1%), central nervous system (4.8%), psychotherapeutic (4.0%), respiratory (1.4%), hematopoietic (0.9%), and topical nasal (0.9%) agents (Table 2). Interaction analysis showed that BMI class influenced the association of VATob with cardiovascular, metabolic, psychotherapeutic, and hematopoietic agents (Table 4) Specifically, normal-weight participants with VATob had a higher risk of being prescribe a cardiovascular, a metabolic, or a hematopoietic agent. Therefore, this study posits that VAT should be measured in normal-weight individuals.

Here, for normal-weight participants, the risk of receiving cardiovascular, metabolic, or hematopoietic agents was enhanced by VATob, more than overweight and obese participants. Therefore, it is posited that VATob is more detrimental in the absence of substantial SAT. Recently, a condition has been described in which subjects with normal BMIs (18.5 to 24.9 kg/m^2^) can have elevated VAT, resulting in the presence of metabolic complications. These “hidden obese” subjects could potentially be missed for treatments [32]. This potentially means that the distribution of body fat could be a greater risk factor than either weight gain or BMI [33]. The American Heart Association stated that the level of misdiagnoses of cardiometabolic disease in individual with “normal-weight obesity”—normal weight individuals with metabolic and inflammation profiles similar to obese individuals—is higher. They even suggest using waist circumference, an index more associated with VAT than SAT, could be more prognostic for cardiovascular disease [34]. This could explain the higher odds ratios for cardiovascular and metabolic agent use among normal-weight participants. The “obesity paradox” provides support of this possibility. The “obesity paradox” is when obese patients have better outcomes than other BMI classifications [35]. Several explanations had been posited; however, the counter-active effects of SAT to VAT are very plausible. Peripheral adiposity confers cardiovascular protection due to the secretion of adiponectin, which has an anti-inflammatory effect [36], in contrast to VAT, which secretes interleukin-6, interleukin-8, angiotensinogen, vascular endothelial growth factor, monocyte chemoattractant protein-1, promoting more chronic inflammation and dyslipidemia [14].

Environmental and lifestyle factors, such as high-calorie diets, sedentary behaviors, psychological stress, and aging, contribute to VAT accumulation. VAT serves as an active lipid storage depot with mesenteric fat fostering a high lipogenic environment due to continuous lipid flux [15, 37]. Its proximity to organs allows VAT to exert localized metabolic effects, such as promoting atherosclerosis and cardiovascular disease [18]. VAT plays a central role in driving systemic inflammation and metabolic dysfunction through its dysregulated secretion of pro-inflammatory adipokines, including leptin, interleukin-6, resistin, tumor necrosis factor-α, and visfatin [38]. This pro-inflammatory milieu activates pathways such as the NF-κB pathway, perpetuating chronic inflammation that underlies insulin resistance, metabolic syndrome, MASLD, and cardiovascular disease [3, 39–41]. The metabolic disturbances associated with VAT accumulation are linked to an increased need for medications targeting these conditions, such as lipid regulators, antidiabetics, and cardiovascular agents [42]. Furthermore, VAT’s influence extends to pharmacokinetics, as its expansion alters drug metabolism by affecting hepatic cytochrome activities (e.g., CYP3A4, CYP2C19) and renal clearance [43], impacting the efficacy of medications like statins [43, 44]. Weight gain and VAT accumulation exacerbate cardiometabolic risk via localized inflammation, immune cell activation, and cytokine release, contributing to higher prescription use [45]. Additionally, medications themselves, such as psychotropics and antidiabetics [46, 47], can disrupt metabolic homeostasis and promote VAT accumulation, creating a feedback loop of inflammation and metabolic dysfunction. Collectively, VAT’s unique inflammatory and metabolic profile provides a stronger predictor of medication needs than BMI alone, underscoring its pivotal role in the pathophysiology of chronic disease management.

Healthcare studies have showed an augmented cost associated with obesity, particularly in patients with Type 2 Diabetes, hypertension, depression, and other chronic conditions [48]. Although no studies specifically address the costs associated with VATob, similar expenses are likely. Harrison *et al*. observed that higher BMI correlated with an increased prevalence of chronic health conditions, which amplifies healthcare costs [49]. A systematic review confirmed that obesity augments healthcare costs, specifically in the USA, obesity-related costs raised from $2.7 billion USD in 2005 to $6.9 billion USD in 2011 [50]. Therefore, it is posited that VATob will augment the cost as well. For that reason, clinicians should focus on reducing VATob as much as weight control. Increasing physical activity is one potential intervention. Our findings demonstrated that higher physical activity levels reduced the likelihood of VATob-associated prescription use. However, this effect was not evident when examining specific medication classes, suggesting the reduction was driven by fewer medications or fewer medication classes overall. Future studies should further investigate this relationship.

Here, 24.4% and 5.8% of the cohort was taking ≥2 or ≥5 prescriptions, respectively. Polypharmacy (use of ≥5 medications) is a critical concern due to its association with adverse outcomes, including increased hospitalizations and mortality [51]. VATob was associated with a greater risk of polypharmacy, particularly among participants classified as overweight or obese class I. While this effect was less evident in the normal-weight and class II obese groups, the limited sample sizes suggest the need for larger studies to confirm these findings. Reducing VAT may help mitigate the risks of polypharmacy and even facilitate discontinuation of certain medications. VAT reduction has been associated with improvements in inflammatory cytokine profiles, insulin levels, and plasminogen activator inhibitor-1 levels [52, 53], as well as promoted longevity [54]. Recently, a study demonstrated that a personalized intervention that included Plant-Based Whole Foods, Time Restricted Eating, and Fractionized Exercise led to a 67% decrease in insulin doses and a 27% reduction in the number of medication categories [29]. However, no studies have specifically assessed the impact of VAT reduction on prescription needs, highlighting an important area for future research.

This study has a few limitations. First, while key factors affecting prescription use were adjusted, other VAT-associated factors, such as smoking and triglyceride levels, were missing. Second, insurance data does not describe the kind of prescription coverage. Different insurances, even from the same provider, can have different types of prescription coverage. Third, smokers were included as well as other lifestyle conditions that affect prescription use [55]. Fourth, the classification of the prescriptions was made according to Vademecum. Other systems could modify the classification, which could move a few medications between categories. Moreover, the reason for the prescription was not considered. Off-script prescription use was not taken into consideration. For example, Ozempic® (semaglutide) can be used for weight loss instead of its glucose-lowering effect. Fifth, prescription use was reported; however, it should be noted that medication compliance and efficacy were not considered. Sixth, VATob was defined using a 100 cm^2^ threshold. Participants with 100 cm^2^ or with 400 cm^2^ were considered the same. Kredel *et al*. showed that increased VAT levels are associated with more inflammation [14]. When stratified by overall obesity, it is possible that in normal-weight-VATob participants had VAT values closer to 100 cm^2^, whereas obese-VATob participants had VAT values closer to 400 cm^2^. Finally, as a cross-sectional study, causal associations cannot be established. Future longitudinal studies should explore how changes in VAT influence medication needs.

## Conclusion

VATob is associated with higher overall prescription use, but mainly with cardiovascular and metabolic agents. This association was higher for normal-weight participants than for overweight and obese participants.

## Supporting information

S1 Table STROBE statement—checklist of items that should be included in reports of case-control studies. (PDF)
